# Innovative binary sorption of Cobalt(II) and methylene blue by *Sargassum latifolium* using Taguchi and hybrid artificial neural network paradigms

**DOI:** 10.1038/s41598-022-22662-7

**Published:** 2022-10-31

**Authors:** Zeiad Moussa, Abeer A. Ghoniem, Ashraf Elsayed, Amenah S. Alotaibi, Asma Massad Alenzi, Sahar E. Hamed, Khaled M. Elattar, WesamEldin I. A. Saber

**Affiliations:** 1 Microbial Activity Unit, Department of Microbiology, Soils, Water and Environment Research Institute, Agricultural Research Center (ID: 60019332), Giza, 12619 Egypt; 2 Botany Department, Faculty of Science, Mansoura University, Elgomhouria St., Mansoura, 35516 Egypt; 3 Genomic and Biotechnology Unit, Department of Biology, Faculty of Science, University of Tabuk, Tabuk, Saudi Arabia; 4 Chemistry Department, Faculty of Agriculture, Damietta University, Damietta, Egypt; 5 Unit of Genetic Engineering and Biotechnology, Faculty of Science, Mansoura University, El-Gomhoria Street, Mansoura, 35516 Egypt

**Keywords:** Biological techniques, Biotechnology

## Abstract

The present investigation has been designed by Taguchi and hybrid artificial neural network (ANN) paradigms to improve and optimize the binary sorption of Cobalt(II) and methylene blue (MB) from an aqueous solution, depending on modifying physicochemical conditions to generate an appropriate constitution for a highly efficient biosorption by the alga; *Sargassum latifolium*. Concerning Taguchi's design, the predicted values of the two responses were comparable to actual ones. The biosorption of Cobalt(II) ions was more efficient than MB, the supreme biosorption of Cobalt(II) was verified in run L_21_ (93.28%), with the highest S/N ratio being 39.40. The highest biosorption of MB was reached in run L_22_ (74.04%), with a S/N ratio of 37.39. The R^2^ and adjusted R^2^ were in reasonable values, indicating the validity of the model. The hybrid ANN model has exclusively emerged herein to optimize the biosorption of both Cobalt(II) and MB simultaneously, therefore, the ANN model was better than the Taguchi design. The predicted values of Cobalt(II) and MB biosorption were more obedience to the ANN model. The SEM analysis of the surface of *S. latifolium* showed mosaic form with massive particles, as crosslinking of biomolecules of the algal surface in the presence of Cobalt(II) and MB. Viewing FTIR analysis showed active groups e.g., hydroxyl, α, β-unsaturated ester, α, β-unsaturated ketone, N–O, and aromatic amine. To the best of our knowledge, there are no reports deeming the binary sorption of Cobalt(II) and MB ions by *S. latifolium* during Taguchi orthogonal arrays and hybrid ANN.

## Introduction

Technological processes of heavy metals sorption depend on adsorption, precipitation, ion exchange, membrane filtration, coagulation-flocculation, etc. Overall, these methods have the demerits of being expensive, non-specific, extremely susceptible to pH, and ultimately futile^1^. However, the biosorption process uses biomass, having certain specific biomolecules, which are proficient to attach ions existing in the wasted liquids^2–4^. The biosorption process usually occurs as a result of the affinity between adsorbate and biosorbent. Generally, biosorption techniques have the advantages of better performance, short operation time, low cost of operation, eco-friendliness, high specificity, and freedom from associated contaminants^5–7^.

The algal biomass is favorable since some algal genera have benefits as biosorbents for the confiscation of toxic metals such as cobalt^8^. Algae are a renewable sort of sorbents, which show diverse sympathies for the subtraction of heavy metals. They are also distributed in the ecosystem, (aquatic, freshwater, marine, and terrestrial environment). Similar to plants, marine algae perform some biochemical processes such as photosynthesis, furthermore, their attributes of biochemistry vary substantially^9^. As well, the majority of algae are microscopic forms, while others showed to be macroscopic uniform and grow up to 100 feet^10^. Interestingly, the annual production of seaweeds exceeds 3 million tons, while the potential harvesting of red algae is 2.6 million tons, with the brown algae production being 16 million tons^11^. Algae produce some biomaterials such as alginate, agar, and carrageenan, therefore have been included in the food industry. Moreover, seaweeds are a potential source of several biological constituents such as phenolics, vitamins, pigments, and dietary fibers, with medications concern, owing to their richness of cell wall polysaccharides^12^. Further, they are also described in folk medicine as therapy for stomach ulcers, heart diseases, asthma, gall stones, and anti-mutagenesis^13^. The alga, *Sargassum latifolium* extracts showed an inhibitory effect against the hypoxia pathway in cells of colon cancer^14^.

Otherwise, the merits of engaging biomass of algae involve sophisticated surface area, availability of biomass, economical aspects, high efficiency, and privileged coercing affinity towards metals^15^. The function of particular algal biomass towards heavy metals is reasonably greater than traditionally available adsorbents *e.g*., activated carbon, artificial resin, and natural zeolite^1,16^. The efficiency of algal biomass in the removal of heavy metals is due to the dispersion of proteins, carbohydrates, and lipids on the cell wall of algae, whereby the functional groups, e.g., amino, amide, phosphate, imidazole, carboxyl, thioether, phenolic, and sulfhydryl, offer the affinity of binding with heavy metals^17^.

Dittert, et al. ^18^ pointed out that the efficient alginic acid content of the cell wall of alga, *Laminaria digitata*, plays a crucial task in removing chromium. Whereas, the functional groups, such as methyl, carboxyl, hydroxyl, sulfate esters, sulfonate, and alcohol that were distributed on the cell wall of *Pelvetia canaliculata* were effective in leaching lead and cadmium ions^19^. Likewise, the alga, *Pelvetia canaliculata*, was active in removing copper and zinc by function groups of carboxylic, and sulfonic^20^. The activity of *Enteromorpha* sp. in removing chromium reached 5.347 mg/g biomass^21^.

The risk of heavy metals is owing to their non-degradable, with accumulation into living tissues. The concentration of heavy metals if exceeding the permissible level could be toxic to ecosystems. Moreover, several diseases are associated with the presence of heavy metals e.g., irritability, nausea, loss of appetite, liver and renal conflicts, bone injury, and muscular stiffness^22,23^. Consequently, the absorptive procedures of heavy metals might be mandatory to reduce metals within permissible limits^24^.

Synthetic dyes are considered environmental pollutants with detrimental concern to humans owing to their discharge into the environment as the industrial activities by mankind^25,26^. The annually huge quantity of synthetic dyes was associated with industries of cosmetics, leather, printing, paper, pharmaceutical industry, etc.^27^. Moreover, the dyes have several characteristics of being non-degradable, resistive in nature, and stable (to heat light, and oxidizing agents)^28^. Methylene blue (MB) is one of the synthetic dyes that belongs to heterocyclic aromatic dye, with the chemical formula of C_16_H_18_N_3S_Cl. It is used in coloring (cotton, and silk), tannin printing, and as a therapeutic agent for healing fungal infections as well. MB has been ensued as virulent to human beings since it is associated with clinical symptoms e.g., vomiting, nausea, irritation, chest pain, anemia, mental disturbance, pale or blue skin, and skin irritation^29^.

Biosorption strategy is generally applied using safe biosorbents for leaching of such pollutants, where several microorganisms have proficiency in leaching of dyes, due to the inherent capacity for biodegradation of dyes as a source of carbon and energy transduction^30^. The marine algae showed to be extremely active biosorbents of such dye pollutants from aqueous solutions^31^. The kinetics of biosorption could be back to the cell wall of algae, by which the active groups e.g., the hydroxyl, carboxyl, amino, and sulfate could be played a vital role in the binding process of dyes pollutant^32^.

The integration of statistical and mathematical procedures with biological principles is one of the major contributions to enhancing the biosorption processes, with feasibility, accuracy, and finally optimizing the inputted independent parameters to maximize the output of the biosorption. The statistical paradigms have been adopted efficiently in the optimization of the biosorption process of heavy metals^33^. The experimental design and statistical procedures depend upon their efficiency in distinguishing the correlation between dependent and independent variables^34^. Additionally, they are effective and powerful tools for choosing the important aspects rapidly from multivariate experiments for optimization of certain process^35^.

Taguchi's approach is a robust fractional factorial design and multiparameter optimization statistical technique. Taguchi design is one of the operative statistical procedures, which categorizes systematic experimentation to scale the optimizing parameters for performance, quality, and sacrifice. One of the major merits is the ability to achieve desired response using compacted experimental runs of several variables under study, depending upon orthogonal arrays^36,37^. Additionally, it is a simple and systematic way of determining the effect and the optimum level of the factors. The arrays of the Taguchi method have evolved as extensions of factorial designs and Latin squares (L) called the orthogonal arrays, which employ various combinations of factors^37,38^. An orthogonal array (more specifically a fixed element orthogonal array) is a matrix whose every pair of columns, each of the possible ordered pairs of factors appears the same number of times^38^. The output of the orthogonal arrays is optimized concerning signal to noise (*S*/*N*) ratio of the responses instead of the responses themselves and thus it reduces the process variability^39^. The S/*N* ratio marks the difference between the conventional statistical technique and the Taguchi method. The S/*N* ratio measures the deviation of the response from the desired value. Where signal implies the mean value while noise implies the standard deviation term. It means that lower variability in the process is ensured by maximizing the *S*/*N* ratio. Depending on the type of response desired, Taguchi classified *S*/*N* ratio into three categories i.e., smaller is better, larger is better, and nominal is better^36,38^. The S/N ratio is a substitute for response itself to govern the optimum situations of control factors and thus ignored the variations caused by the uncontrollable factors^36^.

The artificial intelligence approach occupies a unique position in the current era, artificial neural network (ANN) is the central piece of this approach and one of the main tools applied in machine learning. ANN is an advanced tool that can act like the human brain, through analyzing the patterns, processing the data, and thus building computational models with inter-communicated nodes (neurons) within the hidden layer(s). This intelligence modeling approach enables making accurate choices based on a given investigational data. Through ANN modeling, the network architecture is selected, then constructing the input, hidden, and output layers with enough neurons. Such a network starts learning and training processes until understands the data pattern. Finally, validation and verification of the resulting ANN model take place before approving the predictive model^40,41^. The learning mechanism of ANN is based on diagnosing the diverse designs in the data to detect any differences and decide which pattern achieves the target, this process is controlled through intelligent backpropagation that generates the desired model that attains the goal. The deep learning procedure is supposedly more truthful and can effectively substitute the other modeling policies^6,42^.

The biotechnological process of our present study aims to use the algal biomass of *S. latifolium* to remove both Cobalt(II) ions (sulfate salt was used due to its outbreak in the environment, especially in a case of pollution of water and soils, as well as its high toxicity) and the synthetic dye; MB, in a binary state with the following items in mind; (I) investigation of optimized simultaneous biosorption of Cobalt(II) and MB ions by biomass of *S. latifolium* during paradigms of Taguchi orthogonal array and hybrid ANN, (II) studying the functional groups of the algal cell wall by Fourier-transform infrared, and (III) examination of the cell wall alterations of *S. latifolium* by scanning electron microscopy.

## Materials and methods

### Formulation of dried biomass of alga

The brown alga; *Sargassum latifolium* was kindly gained from the National Institute of Oceanography and Fisheries, Hurghada, Egypt. Algal biomass was washed with deionized water to avoid the accumulation of other ions that may impair the biosorption process. The cleaned algal biomass was dried at 60 °C to protect the chemical components of the algal biomass from cleavage by high temperature. Thalli were then crushed into small pieces and sieved through sieve sizes ranging from 0.5 to 1 mm^43^.

### Cobalt and methylene blue solution

Cobalt sulfate (CoSO_4_.7H_2_O) and methylene blue (MB) were obtained from Sigma-Aldrich, Cairo, Egypt. A standard stock solution from each chemical was prepared by dissolving a known amount in distilled water. The chosen concentrations of both cobalt and methylene blue were conducted by dilution of stock solutions.

### Taguchi experimental design

The simultaneous biosorption experiments of both cobalt and methylene blue dye using *S. latifolium* were conducted in a batched paradigm, in a working volume of 50 ml. The evaluation of the efficiency of simultaneous removal of both Cobalt and methylene blue was carried out, assessing different independent variables (algal biomass (g/100 ml), operative pH, incubation (contact) time (min), initial Cobalt(II) concentration (mg/L), and initial MB concentration (mg/L). The orthogonal array of Taguchi was used to determine the optimum conditions for the biosorption of both Cobalt(II) ion and methylene blue (MB) using the algal biomass of *S. latifolium*. The orthogonal experimental design was prepared with a minimum number of experiments, using 5 factors each of three levels (high, middle, and low), accordingly the generated design, a total of 27 runs were required for the orthogonal array (L_27_). A generic signal-to-noise (S/N) ratio was used to quantify the experimental variation. This method seeks to improve product or process quality by reducing the mean squared deviation. Depending on the current conditions the type of function applied involved was the larger is better, which is used to maximize the biosorption process according to the next Eq. (1):

1 $$S/N \mathrm{ratio}=-10\times \mathrm{log}(\Sigma (1/{Y}^{2})/\mathrm{n})$$

where n is the number of observations and *Y* is the observed data.

The optimized factors were algal biomass (g/100 ml), pH, time of incubation, initial cobalt concentration (mg/L), and initial MB concentration (mg/L). The levels (actual and coded) of the tested factors and the orthogonal design (L_27_) are presented in Table (1). The analysis of variance (ANOVA) was performed to determine which process parameter(s) is statistically significant. The S/N ratio and ANOVA analyses allow the prediction of the optimal combination of process parameters. A confirmation experiment is then conducted to verify the optimal process parameters determined from the parameter design.

### ANN modeling of the biosorption process

For simultaneous modeling of Cobalt(II) ion and MB biosorption, the Taguchi matrix and the data of the two responses (Table 1) were used to feed a fully connected neural network platform. The constructed ANN contained one input layer with five nodes, representing the five tested factors, and one output layer included two nodes, representing Cobalt(II) ion and MB biosorption. A third layer(s) was located between the input and the output layers. To obtain the best architecture structure of ANN, several hidden layer(s) and node(s) were investigated using several activation functions, by examining several layers and neurons at various learning rates. Accordingly, the ANN topology is designated as 5-h-2. The data were portioned and tested at various holdback propagations, randomly, into three datasets, the first for training (to minimize prediction error and establish neural weights, the second for validation (to stop ANN training and selection of the best model), and the third is an external dataset used for testing the robustness and prediction capabilities of the ANN. The latter dataset was excluded during model development and selection. Machin learning continued using trial and error until the highest coefficient of determination (R^2^) was obtained with the minimum values of error, as well as the predicted ANN outputs were extremely close to the real values of both responses.

**Table 1:** Design of Taguchi’s L_27_ (3^5^) orthogonal array for maximization of both Cobalt(II) and MB biosorption, using the biomass of *Sargassum latifolium*, accompanied by the Taguchi-predicted values and their S/N ratio as well as, the ANN-predicted values at each data point.

Run	Tested factors					Cobalt(II) biosorption, %				Methylene blue biosorption, %			
	X1	X2	X3	X4	X5	Actual	Taguchi		ANN	Actual	Taguchi		ANN
							Predicted	S/N ratio			Predicted	S/N ratio	
L1	1	1	1	1	1	78.20 ± 2.25	76.82	37.86	77.92	53.07 ± 1.38	53.22	34.50	54.64
L2	1	1	1	1	2	75.97 ± 0.43	76.90	37.61	77.52	45.06 ± 0.35	45.68	33.08	47.39
L3*	1	1	1	1	3	77.36 ± 1.30	77.81	37.77	76.27	43.04 ± 0.43	42.27	32.68	42.97
L4*	1	2	2	2	1	77.56 ± 0.49	79.39	37.79	77.54	54.16 ± 2.29	53.26	34.67	54.20
L5*	1	2	2	2	2	79.89 ± 0.80	79.47	38.05	80.18	45.07 ± 1.33	45.73	33.08	45.17
L6	1	2	2	2	3	81.80 ± 1.55	80.38	38.26	82.18	42.08 ± 1.31	42.32	32.48	41.49
L7	1	3	3	3	1	82.50 ± 0.31	80.81	38.33	82.43	49.08 ± 0.32	51.17	33.82	49.91
L8*	1	3	3	3	2	81.08 ± 0.45	80.90	38.18	80.45	44.09 ± 1.32	43.64	32.89	44.11
L9	1	3	3	3	3	79.94 ± 1.31	81.81	38.06	80.71	41.87 ± 1.28	40.23	32.44	41.63
L10*	2	1	2	3	1	81.78 ± 2.19	82.25	38.25	82.77	64.17 ± 1.22	64.58	36.15	64.19
L11	2	1	2	3	2	82.04 ± 0.37	82.33	38.28	82.70	57.07 ± 0.28	57.05	35.13	58.00
L12*	2	1	2	3	3	84.00 ± 1.13	83.24	38.49	83.82	54.03 ± 1.23	53.64	34.65	54.07
L13*	2	2	3	1	1	88.02 ± 0.48	87.09	38.89	88.87	61.05 ± 0.39	61.55	35.71	61.21
L14*	2	2	3	1	2	87.53 ± 0.33	87.17	38.84	88.04	54.14 ± 2.26	54.02	34.67	54.01
L15*	2	2	3	1	3	86.79 ± 0.34	88.08	38.77	86.38	50.98 ± 0.40	50.60	34.15	51.12
L16*	2	3	1	2	1	83.45 ± 0.44	83.48	38.43	83.40	65.07 ± 0.44	63.96	36.27	65.14
L17*	2	3	1	2	2	84.00 ± 0.42	83.56	38.49	83.83	56.28 ± 0.37	56.43	35.01	56.27
L18*	2	3	1	2	3	84.06 ± 1.23	84.47	38.49	84.15	52.06 ± 1.22	53.02	34.33	52.03
L19*	3	1	3	2	1	90.83 ± 1.39	91.24	39.16	90.57	73.04 ± 0.35	72.56	37.27	73.10
L20*	3	1	3	2	2	90.68 ± 0.37	91.32	39.15	90.83	66.08 ± 0.36	65.03	36.40	65.93
L21*	3	1	3	2	3	93.28 ± 0.26	92.23	39.40	92.96	60.08 ± 1.28	61.61	35.57	60.03
L22*	3	2	1	3	1	86.06 ± 1.43	87.13	38.70	85.34	74.04 ± 1.27	73.88	37.39	74.08
L23	3	2	1	3	2	87.07 ± 2.43	87.21	38.80	86.28	66.05 ± 1.25	66.34	36.40	68.05
L24*	3	2	1	3	3	89.34 ± 1.33	88.12	39.02	89.03	63.06 ± 1.31	62.93	36.00	63.05
L25	3	3	2	1	1	91.19 ± 0.35	91.38	39.20	91.85	73.03 ± 0.23	72.52	37.27	71.24
L26	3	3	2	1	2	92.08 ± 1.32	91.47	39.28	92.06	65.06 ± 0.26	64.98	36.27	64.93
L27*	3	3	2	1	3	91.96 ± 1.35	92.38	39.27	91.76	60.98 ± 0.38	61.57	35.70	61.04

Factors and levels employed in Taguchi’s experimental designs matrix													
Tested Factor		Level											
Symbol	Actual name	Low (1)	Middle (2)	High (3)									
X1	Algal biomass (g/100 ml)	0.3	0.6	0.9									
X2	pH	6.5	7.5	8.5									
X3	Incubation time (min)	30	60	90									
X4	Initial Cobalt(II) (mg/L)	50	100	150									
X5	Initial methylene blue (mg/L)	15	20	25									

*The 18 runs that were used for training of the network, the other runs were used for the validation process.

### Testing and validation of models

Upon the determination of the prediction models, the fitness of Taguchi and ANN models were then tested and compared through residual analysis by the determination of root mean square error (RMSE) and mean absolute deviation (MAD). Finally, both Taguchi and ANN models were experimentally validated based on the optimum levels of the five factors calculated from the prediction models.

### Software and statistical examination

The experimental results of the biosorption of Cobalt(II) ion and methylene blue by *S. latifolium* are expressed as the mean ± standard deviation (SD) of three biological replicates. Taguchi orthogonal array and its statistical analysis were accomplished using Minitab statistical analysis software package (Minitab Inc. version 21, USA, https://www.minitab.com/en-us/). The machine learning and ANN topology for modeling multi-response optimization were set up using the JMP Pro statistical analysis software package (JMP pro.®, Version 16.2, SAS Institute Inc., Cary, NC, https://www.jmp.com/en_us/home.html).

### Analytical procedures

The content of each flask for the Taguchi experiment was centrifuged at 6000xg and analyzed for residual cobalt(II) using atomic absorption spectroscopy according to standard methods for the examination of water and wastewater 23rd edition, 2017^44^. The residual methylene blue was measured spectrophotometrically at 670 nm. The capacity of *S. latifolium* biomass as biosorbent for removal of Cobalt(II) or residual methylene blue was determined using the same Eq. (2) as follows:

2 $$\mathrm{Removal\, efficiency \%}=\mathrm{C}1-\mathrm{C}2/\mathrm{C}2$$

where C1 and C2 are the initial and residual concentrations of methylene blue or cobalt.

### Scanning electron microscopy (SEM) of *S. latifolium* cell wall

The cell surface of alga, *S. latifolium*, was coated with gold and examined before and after the biosorption process by SEM (SEM-2100, JEOL, Tokyo, Japan) attached to an accelerating voltage of 20 kV at the Central Laboratory, Electron Microscope Unit, Mansoura University, Egypt.

### Fourier-transform infrared (FTIR) spectroscopy

FTIR analysis was performed to illuminate the distinct surface functional groups of *S. latifolium* biomass that could be accountable for the binding of cobalt and MB. The samples of biomass of alga were analyzed before and after the biosorption process, with FTIR spectroscopy (Thermo Fisher Nicolete IS10, USA, spectrophotometer). The dry biomass sample of *S*. *latifolium* was mixed with pellets of potassium bromide, then FTIR spectra were analyzed within the range of 400–4000 cm^−1^.

## Results and discussion

In common, the large scale of the industry with mass productivity has benefits for human beings’ society, with a variety of services and products, whereas the massive quantity of contaminants has been generated such as heavy metals and synthetic pigments (phenol and methylene blue dyes). These chemical pollutants were considered as high toxicity, especially to human beings and the ecosystem, as well^45^. Moreover, these chemicals were pondered as urgent pollutants corresponding to the US Environmental Protection Agency^46^. Otherwise, many species of seaweeds showed to accumulate a high-level concentration of heavy metals, consequently, they could be indices of metals pollutants in the environment^47^.

Methylene blue (MB) is one of the synthetic dyes that belongs to heterocyclic aromatic dye, with the chemical formula of C_16_H_18_N_3S_Cl. It is used in coloring (cotton, and silk), tannin printing, and as a therapeutic agent for healing fungal infections as well. MB has been ensued as virulent to human beings since it is associated with clinical symptoms e.g., vomiting, nausea, irritation, chest pain, anemia, mental disturbance, pale or blue skin, and skin irritation^48^.

As well as marine algae species have priority in biosorption of dyes pollutants, whereby the biosorption occupied onto cell surface of algae by functional groups^49^. *Sargassum* is a genus of brown algae under the family of Sargassaceae, containing approximately 400 species that are consumed as food and herbal medicine since they are rich in bioactive compounds e.g., fucoidans, sterols, glycolipids, and meroterpenoid, so it could be used in many medical, and biotechnological trends^50^.

The previous works involved the capability of algae in leaching several heavy metals and dyes. Novelty, the present controversial study entails the biosorption process of cobalt and MB dye using *S. latifolium* biomass. The process was scaled up using the Taguchi paradigm to evaluate the independent variables that influence the biosorption process. Then, a hybrid artificial neural network was performed to order to simultaneously optimize the biosorption process of both Cobalt(II) and MB dye.

Concerning Taguchi design, the biosorption process was tested against 5 parameters (algal biomass, %, pH, time of incubation, initial cobalt concentration, mg/L, and initial MB concentration, mg/L), each at three levels (3^5^). The orthogonal array of Taguchi design indicated that a total of 27 runs were required for maximization of biosorption of both Cobalt(II) and MB, using the biomass of *S. latifolium*. To our knowledge, dual optimization of cobalt biosorption by *S. latifolium* biomass using Taguchi experimental designs was never reported. The optimization process by Taguchi experiments often starts with the determination of the S/N ratio to identify those control factors that reduce variability. Next, identify control factors that move the mean to target and have a small, or no effect on the S/N ratio. Besides saving time and effort, the orthogonal array of Taguchi design makes it possible to develop satisfactory conditions using minimum experimental runs^36^. That is why the Taguchi method was utilized here to identify the optimal conditions that boost the biosorption process.

The orthogonal arrays of the L_27_ (3^5^) type and the corresponding biosorption percentages of both Cobalt(II) and MB data recovered from the laboratory implementation of the 27 runs, as well as the predicted values based on the Taguchi model, are depicted in Table (1). First, the data of both responses were checked for the ability to be modeled by the Taguchi array method. The predicted values for both responses were very close to the actual ones, indicating the accuracy of the models’ prediction. Data displayed that algal biomass absorbed Cobalt(II) more efficiently than MB. The S/N ratio of each data point of the design was also introduced, where the maximum biosorption of Cobalt(II) (93.28%) was achieved by the run L_21_, which also recorded the highest S/N ratio (39.40), compared with the highest run (L_22_) in MB biosorption (74.04%), and its S/N ratio (37.39).

**Figure 1:**
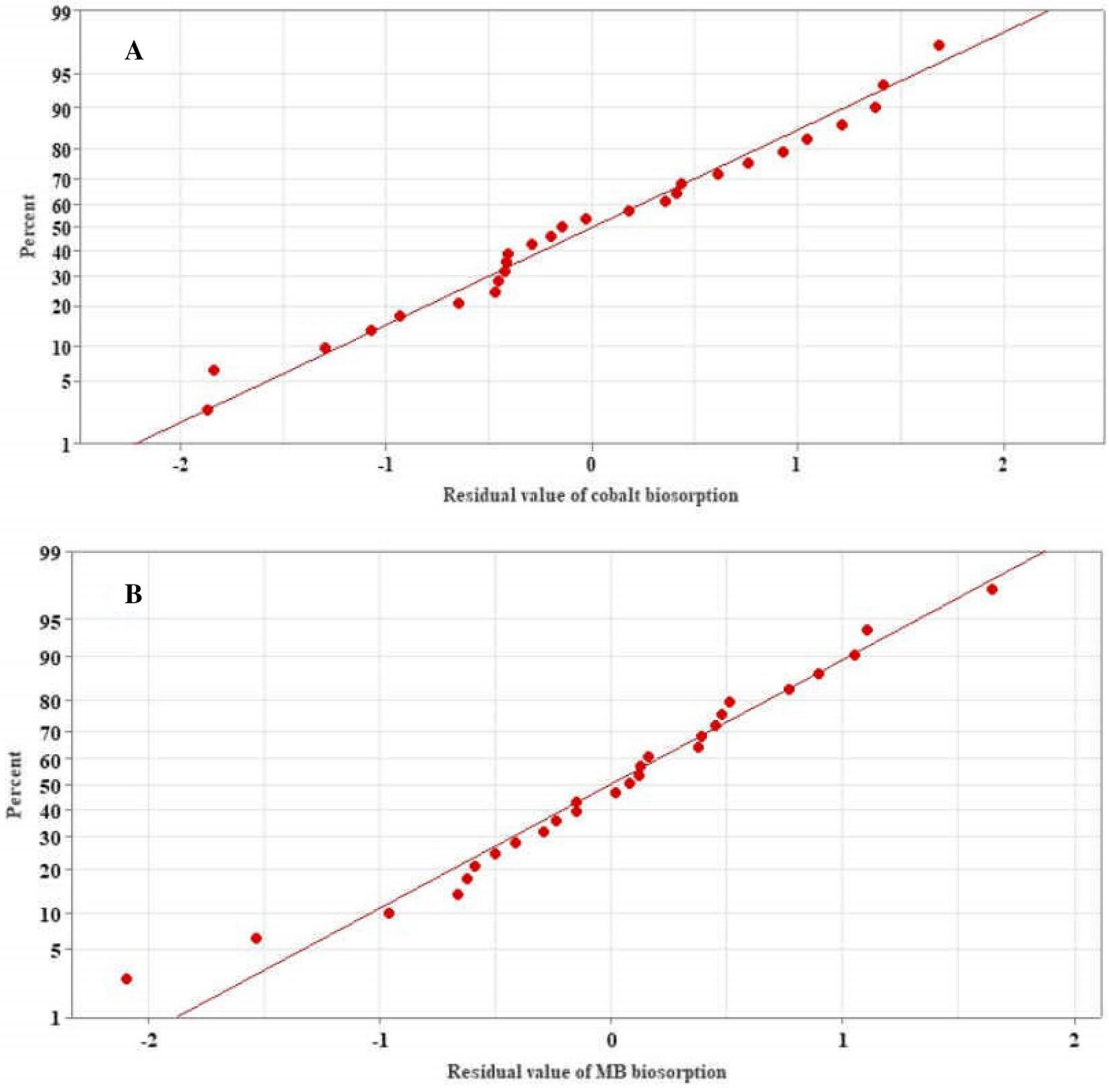
Normal probability plot of residuals of cobalt biosorption (**A**) and MB biosorption (**B**) by the biomass of *Sargassum latifolium*.

The ability of Taguchi design to model the data of biosorption of both Cobalt(II) and MB was evaluated. The residual (difference between actual and predicted value) analysis was investigated by plotting the normal probability plot of residuals (Fig. 1A,B). The pattern of the normal probability plot of the residuals for the two responses approximately follows a straight line, and the residual point was very close to the straight line of best fit, assuming the normal distribution of the residuals and the suitability of the data for modeling both Cobalt(II) and MB biosorption.

Moreover, additional model statistics were calculated (Table 2). The standard deviation of both responses (Cobalt(II) and MB biosorption) was found to be very low; this is a good indicator since standard deviation is measured in the units of the response variable and represents how far the data values fall from the fitted (predicted) values. The lower the value of standard deviation, the better the model describes the response. However, a low standard deviation value by itself does not indicate that the model meets its assumptions, consequently, the previous residual analysis with the aid of standard deviation confirmed the assumptions of the model.

**Table 2:** Model summary statistics and ANOVA of both Cobalt(II) and MB biosorption, using the biomass of *Sargassum latifolium* as affected by the tested factors.

**Model summary statistics**					
Indicator	Cobalt(II) biosorption, %	MB biosorption, %			
Standard deviation	1.2168	1.0283			
R^2^	0.9642	0.9932			
Adjusted-R^2^	0.9419	0.9890			

ANOVA for means of Cobalt(II) biosorption, %					
Source	Freedom degree	Sum of square	Mean square	F-value	P-value
Alga biomass (%)	2	535.8	267.9	181.0	0.000*
pH	2	14.7	7.3	5.0	0.021*
Time (min)	2	68.6	34.3	23.2	0.000*
Initial cobalt (mg/L)	2	14.2	7.1	4.8	0.023*
Initial MB (mg/L)	2	5.5	2.7	1.9	0.190
Residual error	16	23.7	1.5		
Total	26	662.6			
**ANOVA for means of MB biosorption, %**					
Alga biomass (%)	2	1881.0	940.5	889.5	0.000*
pH	2	3.7	1.9	1.8	0.203
Time (min)	2	19.9	9.9	9.4	0.002*
Initial cobalt (mg/L)	2	3.9	2.0	1.9	0.187
Initial MB (mg/L)	2	564.8	282.4	267.1	0.000*
Residual error	16	16.9	1.1		
Total	26	2490.3			

R^2^ is the coefficient of determination, * indicates a significant effect.

The determination coefficient (R^2^) and adjusted-R^2^ were also calculated. Both are other model evaluation criteria. The present R^2^, and adjusted R^2^ values are close to one, being 0.9642 and 0.9932 and 0.9419 and 0.9890, respectively. Both kinds of R^2^ rang from zero to one. Generally, the closer to 1, the greater the modeling capacity of the data^51^. If their values are 0.9 or greater, the model is regarded as adequate and highly significant, however, R^2^ should not be lower than 0.75^52,53^. R^2^ value measures the change in response (Cobalt(II) and MB biosorption) that results from the variation in the amount of the tested factor(s), irrespective of the significance of the factors, increasing the number of predictors (factors) leads to a constant increase in the R^2^ value. As a result, the adjusted-R^2^ is a modified version of R^2^ that takes into account the number of model factors. In contrast to R^2^, the adjusted R^2^ changed wisely when adding additional only significant factor(s) to the model. As a result, adjusted-R^2^ is a better indicator than R^2^ for determining model fitness. The predicted values of Cobalt(II) and MB biosorption calculated based on the Taguchi model were very close to those of the experimental ones; consequently, the residuals or errors were low, representing another evidence of the accuracy of the model.

To check the significance of each of the tested factors, the data recovered from the Taguchi matrix for both factors were exposed to ANOVA (Table 2). To determine which factor(s) have statistically significant effects on the two responses, the value of probability (P) for each of the five terms was assessed as a diagnosing tool for measuring the significance of the factors. Commonly, the null hypothesis assumes that the term's coefficient is equal to zero, implying that there is no association between the term and the response. The term (factor) is considered significant if it has a lower P-value (< 0.05). A significance level of < 0.05 indicates a 5% risk of concluding that an association exists when there is no actual association. in this connection, for Cobalt(II) biosorption, all control factors had a significant effect except the initial MB concentration. On the other side, the Alga biomass, incubation time, and initial MB concentration have the highest significant variables on MB biosorption. The low P-value, together with the high *F*-value the significance of the suggested overall model^52^.

**Figure 2:**
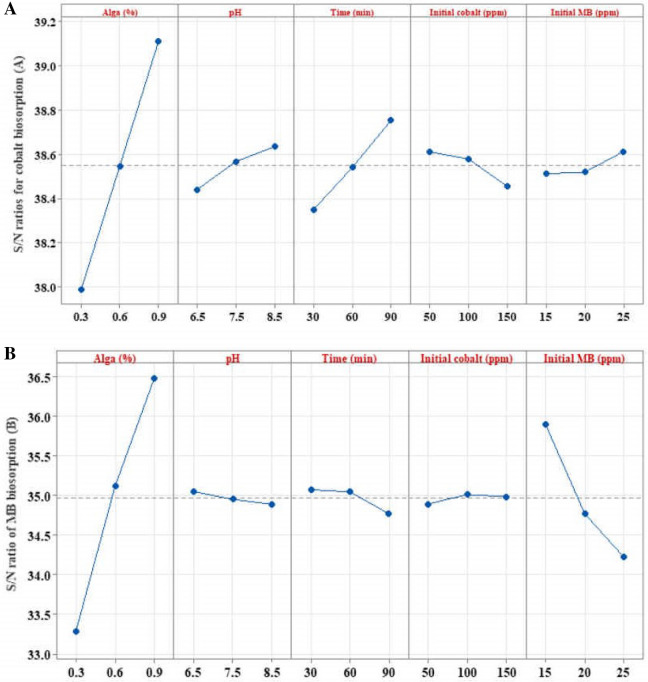
The main effects plot of data means of S/N ratios for cobalt biosorption (**A**) and MB biosorption (**B**) by the biomass of *Sargassum latifolium*, optimized based on larger is better.

Following the previous model checking, Taguchi orthogonal arrays of both factors were used to identify the optimum operating conditions of the parameters that have the most significant effect on the two target outputs. For such purpose, the process parameter’s goal was optimized to a larger S/N ratio that is better for the desired outcome. Accordingly, the best level for each control factor that minimizes the variability caused by the experimental noise was identified. The average S/N ratio was calculated for each level of each factor (Fig. 2). For Cobalt(II) biosorption, the maximum S/N ratio varied for each control factor of the two tested outcomes. Being at algal biomass at 0.9% was the best level for both Cobalt(II) and MB biosorption, whereas the other factors were 8.5 and 6.5 for pH, 90 and 30 min of incubation time, 50 and 100 mg/L of initial Cobalt(II) concentration, and 25 and 15 mg/L of initial MP concentration, respectively.

The Taguchi approach is applied to analyze the mean response for each run in the inner array and to analyze the variation using the S/N ratio, which is different accordingly. The S/N ratio measures how the response varies relative to the target value under different noise conditions. Because of the robust nature of Taguchi design, it can easily identify control factors (five in our case) that reduce variability in a process by minimizing the effects of noise factors that are uncontrollable during the operation. The philosophy of Taguchi designed is to manipulate the noise factors during experimentation to force variability to occur, by this way it could be easy to identify optimal control factor settings that make the process resistant, or robust to variation from the noise factors. All higher ratio of S/N means that the control factor settings are optimum in minimizing the effect of the noise factors^37,54,55^.

The means response analysis for Cobalt(II) and MB biosorption was performed based on Taguchi orthogonal array (Table 3). The results of Cobalt(II) biosorption show that algal biomass (delta 10.91, rank 1) has the largest effect on the mean response, followed by incubation time (delta 3.90, rank 2), then followed by pH, Initial Cobalt(II), and initial methylene blue. Regarding MB biosorption by *S. latifolium*, algal biomass (delta 20.43, rank 1) also has the largest effect on the mean, followed by initial methylene blue concentration (delta 10.95, rank 2), then followed by incubation time, pH, and initial Cobalt(II) concentration.

**Table 3:** Means response analysis of Taguchi array for Cobalt(II) and MB biosorption % by the biomass of *Sargassum latifolium*.

Response for means of Cobalt(II) biosorption, %					
Level	Algal biomass (%)	pH	Incubation time (min)	Initial Cobalt(II) (mg/L)	Initial methylene blue (mg/L)
1	79.37	83.79	82.83	85.46	84.40
2	84.63	84.90	84.70	85.06	84.48
3	90.28	85.58	86.74	83.76	85.39
Delta	10.91	1.79	3.90	1.70	0.99
Rank	1	3	2	4	5

**Response for means of MB biosorption, %**					
Level	Algal biomass (%)	pH	Incubation time (min)	Initial Cobalt(II) (mg/L)	Initial methylene blue (mg/L)
1	46.39	57.29	57.53	56.27	62.97
2	57.21	56.74	57.29	57.10	55.43
3	66.82	56.39	55.60	57.05	52.02
Delta	20.43	0.9	1.92	0.83	10.95
Rank	1	4	3	5	2

Moreover, means response analysis shows that the optimum level of each factor for both responses followed the same trend and came in line with the previous analysis of the S/N ratio, consequently, both analyses confirm each other. Delta value is the difference between the highest and lowest average response values within the factor levels. The ranking is assigned based on delta values; the highest delta value indicates the relative effect of each factor on the response.

The experimental design of Taguchi is a robust design that acts as the central theme of the Taguchi approach to achieve a predictive knowledge of a complex multi-variable process with the fewest possible trials of the experimental process^37,54,55^. The current work reported the suitability of the Taguchi approach to determine the best combination of inputs of five factors that maximize the target output puts (Cobalt(II) and MB biosorption).

Based on the previous results, the five studied factors displayed significant effects with various degrees on Cobalt(II) and MB biosorption. So, the null hypothesis was rejected, and the alternative hypothesis suggests the presence of significant variation due to the five parameters.

### Dual modeling of the biosorption process by ANN

Although the study of Taguchi modeling concluded with the main aim achieved, another drawback of our results arises. Where the optimum biosorption conditions were not the same and varied for each response (Cobalt(II) and MB biosorption). From the practical point of view, it is better to find out only one set of optimum biosorption conditions for both responses. The next trial was to find out the optimum points of the five factors that maximize the biosorption of Cobalt(II) and MB at the same time. For such a target, ANN was the candidate protocol.

The ANN approach of artificial intelligence has found its way into the optimization of biological processes and has emerged as an alternative genius tool for non-linear multivariate modeling^6,40,41^. The prettiness of ANNs as empirical modeling is owing to their capability to accurately extract trends between input and output variables, regardless of the degree of nonlinearity^56^. ANN has the aptitude to acquire knowledge from data, without a previous description of the suitable fitting function, and ANN has entire estimate capability i.e., guessing almost all sorts of non-linear functions including quadratic ones^40,41^. Generally, the ANN required a much greater number of experimental trials to assemble an efficient model. But in fact, ANN can also perform thoroughly even with fairly fewer data^6^.

The response data of the Taguchi array (Table 1) were used to develop a single model for both Cobalt(II) and MB biosorption, employing a fully interconnected multilayer feed-forward ANN. For such reason, numerous hidden layer(s) and neurons within the hidden layer(s) were tested at various combinations of ANN-specific parameters, learning rates, and activation functions.

The ANN training and validation processes were performed using several learning trials, each of 3000 tours, with the trial-and-error procedure, until gaining the best architecture of ANN. Consequently, the best ANN combination parameters were generated to be at a learning rate of 0.1, using the squared penalty method, employing the validation method of a holdback portion of 0.33 (18 runs for training and 9 runs for the validation process). That is, one-third of the data was held out of the model building for validation.

**Figure 3:**
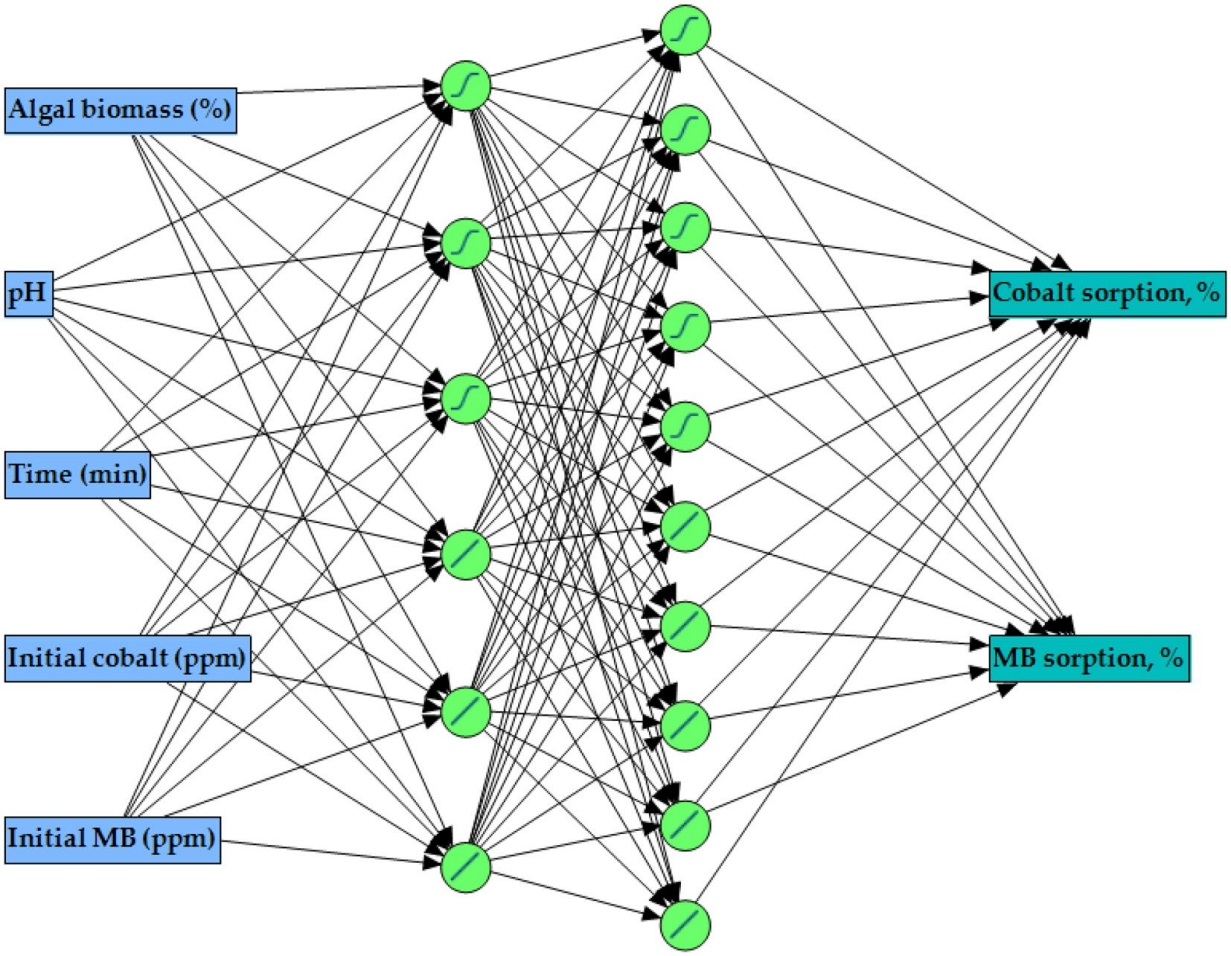
The general layout of the generated artificial neural network for cobalt and MB biosorption by *Sargassum latifolium*, shows an input layer with five neurons, the two hidden layers with 10 and 6 neurons, having a hybrid of hyperbolic tangent sigmoid function (*S*) and linear function (/), and an output layer with two neurons (responses).

The best ANN topology contains one input layer with 5 neurons (five tested factors), and one output layer with two neurons (Cobalt(II) and MB biosorption). In between the input and output layers, two hidden layers were located with two hybrid activation functions. In the first hidden layer, 10 hidden neurons shared equally hybrid activation functions *i.e*., 5 nodes have a hyperbolic tangent sigmoid function (NTanH) and 5 nodes have a linear function (NLinear). The second hidden layer in the ANN has 6 hidden neurons which also shared equally the same hybrid activation functions, three neurons each (Fig. 3). Accordingly, the ANN topology was constructed and designated as 5–6(h)–10(h)-1, with the hybrid model of NTanH(5)NLinear(5)NTanH2(3)NLinear2(3). These conditions are accompanied by the supreme ability of the constructed ANN to predict outputs values of Cobalt(II) and MB biosorption that are very close to the experimental responses.

The artificial intelligence-based approach is an advanced modeling procedure. Despite artificial intelligence has made its way into various applications, there is a shortage of the dual modulation of heavy metals and pigments biosorption by ANN. This is the first work that supports this kind of modeling. Generally, the ANN is flexible enough to generate efficient and accurate models from any kind of response surface, using enough hidden nodes and layers. The function applied at the nodes of the hidden layers is called the activation function^6,42^.

The current ANN platform employed an algorithm of a fully connected multilayer perceptron that perfectly predicts the two response variables using two activation functions at the nodes of the hidden layers. Neural networks employ activation functions in each node, NLinear generates a linear model whereas, to allow the models to be nonlinear, the sigmoid function of NTanH is commonly used. The hybridization between the two activation functions applied here helped the network to learn the complex relationship between inputs (five tested factors) and outputs (Cobalt(II) and MB biosorption).

The current two intermediate (hidden) layers managed a unique correlation between inputs and outputs, therefore playing a central role in correlating the independent variables with the response variables. Therefore, ANN is considered an excellent predictor when it is not necessary to describe the functional form of the response surface or the relationship between the inputs and the outputs^6,42,56^.

Based on the preceding models, the ANN predicted values of each resulting experimental point of both Cobalt(II) and MB biosorption were calculated and given along with the predicted Taguchi values in Table (1). The ANN predicted values show reasonable agreement with the experimental ones. However, the values of R^2^ recorded 0.9879 and 0.9810 (training), and 0.9999 and 0.9834 (validation) for Cobalt(II) and MB biosorption, respectively. Such values are high enough to indicate the success of both the training and validation processes of ANN. As discussed earlier, R^2^ evaluates the correlation between the response and predicted values; therefore, a larger value (up to 1) indicates a significant connection between the input(s) and output(s).

**Figure 4:**
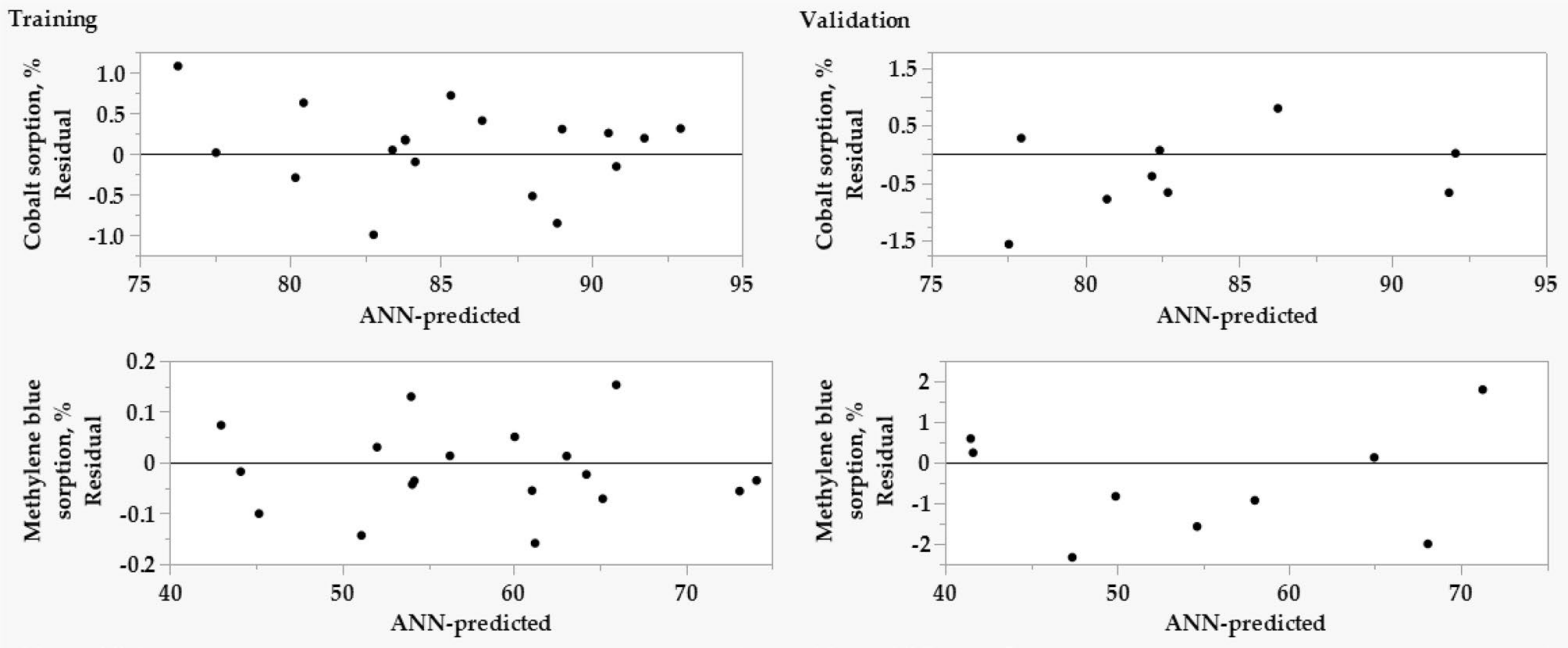
The plots of residuals versus predicted values of both training and validation processes of Cobalt(II) and MB biosorption by the biomass of *Sargassum latifolium*.

The models were also checked by residual analysis to verify their forecasting skills. Plotting the residuals versus predicted values for both training and validation processes of the two responses (Fig. 4) indicates that the residuals are distributed randomly and evenly along the two 0-axis sides, indicating another proof of the success of both training and validation processes.

### Testing and validation of Taguchi and ANN models

Some statistical parameters were calculated to measure and compare the accuracy of the overall models of Taguchi and ANN (Table 4). The values of R^2^ for both models were high, however, the ANN model performed better than Taguchi, especially regarding Cobalt(II) biosorption. The same trend was observed for the error analysis showed that the RMSE and MAD recorded low error values. RMSE is commonly employed in regression analysis to authenticate experimental results since a lower value indicates that the data are concentrated around the line of best fit. MAD is another statistic that determines the average dispersion of data around the mean. A lower MAD value implies a reduced spread of data around the mean. The current conclusion concedes with a recent study^6^, which reported that the lower values of RMSE, and MAD, and higher R^2^ values are good indicators of the fitness of the model.

**Table 4:** Statistics of and performance of Taguchi and ANN models for Cobalt(II) and MB biosorption by the biomass of *Sargassum latifolium*.

		Taguchi predictor		ANN predictor	
		Cobalt(II) sorption, %	MB sorption, %	Cobalt(II) sorption, %	MB sorption, %
**Model statics**					
R^2^		0.9642	0.9932	0.9858	0.9931
RMSE		0.9373	0.7908	0.5912	0.7990
MAD		0.7741	0.6067	0.4603	0.4305
Frequency		27	27	27	27
**Validation of the model**					
Predicted level	Algal biomass (%)	0.9	0.9	0.9	
	pH	8.5	6.5	7.44	
	Incubation time (min)	90	30	79.85	
	Initial Cobalt(II) (mg/L)	50	100	50	
	Initial MB (mg/L)	25	15	15	
Predicted response		94.42	74.49	94.55	74.48
Experimental response		92.78 ± 1.36	72.11 ± 0.76	95.31 ± 0.57	75.07 ± 0.44

RMSE is the root mean squared error, and MAD is the mean absolute deviation.

### Validation of both models

The theoretical values of the optimal combination of the tested variables and the corresponding maximum response of Cobalt(II) and MB biosorption by the biomass of *S. latifolium* were calculated in Table 4. These estimates were evaluated under laboratory conditions to check the forecast capacity of both models. Taguchi model had two separate sets of factors’ levels, and can only predict one response at a time, Cobalt(II) or MB biosorption. On the opposite side, ANN had only one set of conditions for simultaneous modeling of both responses. Moreover, the experimental values of Cobalt(II) (95.31 ± 0.57) and MB (75.07 ± 0.44) biosorption obtained based on the estimated levels by the ANN model were found to be closer and obey to predicted ANN values Cobalt(II) (94.55) and MB (74.48) than those predicted by Taguchi model. However, both models performed better in general. The obvious advantage of ANN is its ability to use similar conditions for simulations biosorption of Cobalt(II) and MB. Such a result, truly, confirms the higher accuracy and predictive ability of both models, however, despite the effectiveness of the Taguchi method in the modeling process, it cannot find similar conditions for Cobalt(II) and MB biosorption.

Although the superiority of ANN as a multi-response optimizer in the current study, it is fair to acknowledge that ANN modeling consumed extended computational time through many iterative calculations. Furthermore, the ANN structured nature cannot demonstrate the contributions and the significance of each factor in the model as Taguchi did, thus the non-significant factors in the model cannot be reduced or eliminated from the model^6,57^. On the other side, ANN had high predictive precision due to its universal ability to approximate the system’s nonlinearity, compared with the other models, which require only a sole step calculation for a response surface model^6,42^.

As shown from the aforementioned data during modeling of the biosorption of Cobalt(II) and MB, a crucial role of the independent variables in the biosorption process could be noticed. A similar role was reported in previous studies. The biosorption of cobalt(II) ions on the biomass of brown alga *Sargassum* sp. using Mg(NO_3_)_2_ as pretreatment was investigated; the optimum biosorption conditions were studied as Mg-treated biomass, temperature 45 °C, initial pH 7.0, biosorption dose 0.1 g, initial concentration of cobalt (II) 300 mg/l, the biosorption capacity of Mg-treated biomass for cobalt (II) ions was 80.27 mg/g, the contact time of biosorption 90 min^58^. The algal biomass of the brown alga *Cystoseria indica* had an impact on simultaneous biosorption of Cobalt(II) and nickel ions; the optimum biosorption process was at pH, 5.9; initial concentration of nickel, 91.94 mg/l; initial concentration of cobalt 89.36 mg/l.; biomass dosage, 0.06 g, sorption time at 80 min, and the maximum biosorption of both nickel and cobalt ions together at the optimum conditions was 69.99 and 75.21 mg/g, respectively (Khajavian et al.^33^. In another study, the brown alga *Sargassum polycystum* was tested for the biosorption of heavy metals, cadmium (Cd) and zinc (Zn); for Cd biosorption were, biosorbent mass: 1.8 g/L, pH: 4.65 and shaking speed: 76 rpm. For Zn, the optimum values were biosorbent mass: 1.2 g/L, pH: 5.7, and shaking speed: 125 rpm, respectively. The maximum value of Cd and Zn uptakes were 105.26 mg/g and 116.2 mg/g respectively^59^.

Further, the biosorption of metals could be managed by the pH values of the solution, which could affect the protonated and deprotonated functional groups. The reduction of metals biosorption in low pH values could be due to competition among binding sites, cations, and the products of acid hydrolysis^60^. In the current investigation, the studied pH range (6.5–8.5) provided the neutral, weak basic, and weak acidic media, which is very common in polluted water and is also suitable for algal biomass. As well, the amphoteric nature of the cell wall of algae can play a vital role in the biosorption process of heavy metals ^1^. Viewing initial doses of metals, other investigations pointed out the overdose of metal in the aqueous solution overcomes the algal biomass resistance, consequently, increasing the algal capacity to absorb additional metal ions^1^. Contrarily, no further significant increase of biosorption has occurred with an increased dose of metals^62^. Commonly, it is difficult to compare our results with the other findings concerning biosorption process of heavy metals and synthetic dyes, owing to different parameters affecting the sorption process, e.g., surface area, proteins, carbohydrates composition, surface charges capacity, etc.^63^ Additionally, the biosorption of MB with its response to independent variables of pH, initial concentration of dye, and contact time has been investigated^64^. Likewise, the proficiency of alga, *Sargassum muticum*, and biomass in biosorption of MB and lead had been studied simultaneously under the various parameters of pH, and initial doses of the algal biomass doses^32^. Li, et al. ^29^ stated the efficiency of activated biochar of *Enteromorpha prolifera* in biosorption of MB dye.

The biomass of brown algae *Sargassum duplicatum* bioapsorbed MB dye. The optimum adsorption was reported at pH 5, initial concentration of MB at 20 mg/L, and adsorbent dosage of 1 g/L. The maximum percentage of dye biosorption was 88,9% ^65^. The biomass of brown alga, *Bifurcaria bifurcata*, had the ability of biosorption of reactive Blue 19 (RB19) and MB dyes from aqueous solutions at optimum pH values were 5.6 and 1.0 for MB and RB19, respectively, Maximum biosorption capacities are 274.45 mg/g for MB and 88.7 mg/g for RB19^66^.

The biomass of brown alga *S. latifolium* had the ability of biosorption of MB and Congo Red (CR) from single and binary systems. Under the optimized conditions, the maximum MB removal was 92.54% and 94.97% for CR for the single system while for the binary system the maximum biosorption was 97.16% and 87.66% for MB and CR, respectively^67^. The biomass of *Sargassum muticum* could bioabsorp up to 93% of MB dye and the concentration and treatment of CaCl_2_ affected the biosorption process^68^.

The biomass of brown alga *Sargassum muticum* the ability of biosorption of MB dye and lead (Pb(II)) from an aqueous medium. The optimum conditions were; 39 mg L^−1^ for MB and 30 mg/ L for Pb(II) ions, contact time (30 min), and brown alga mass (0.3 g)^32^.

### The surface topology of *S. latifolium* by SEM

**Figure 5:**
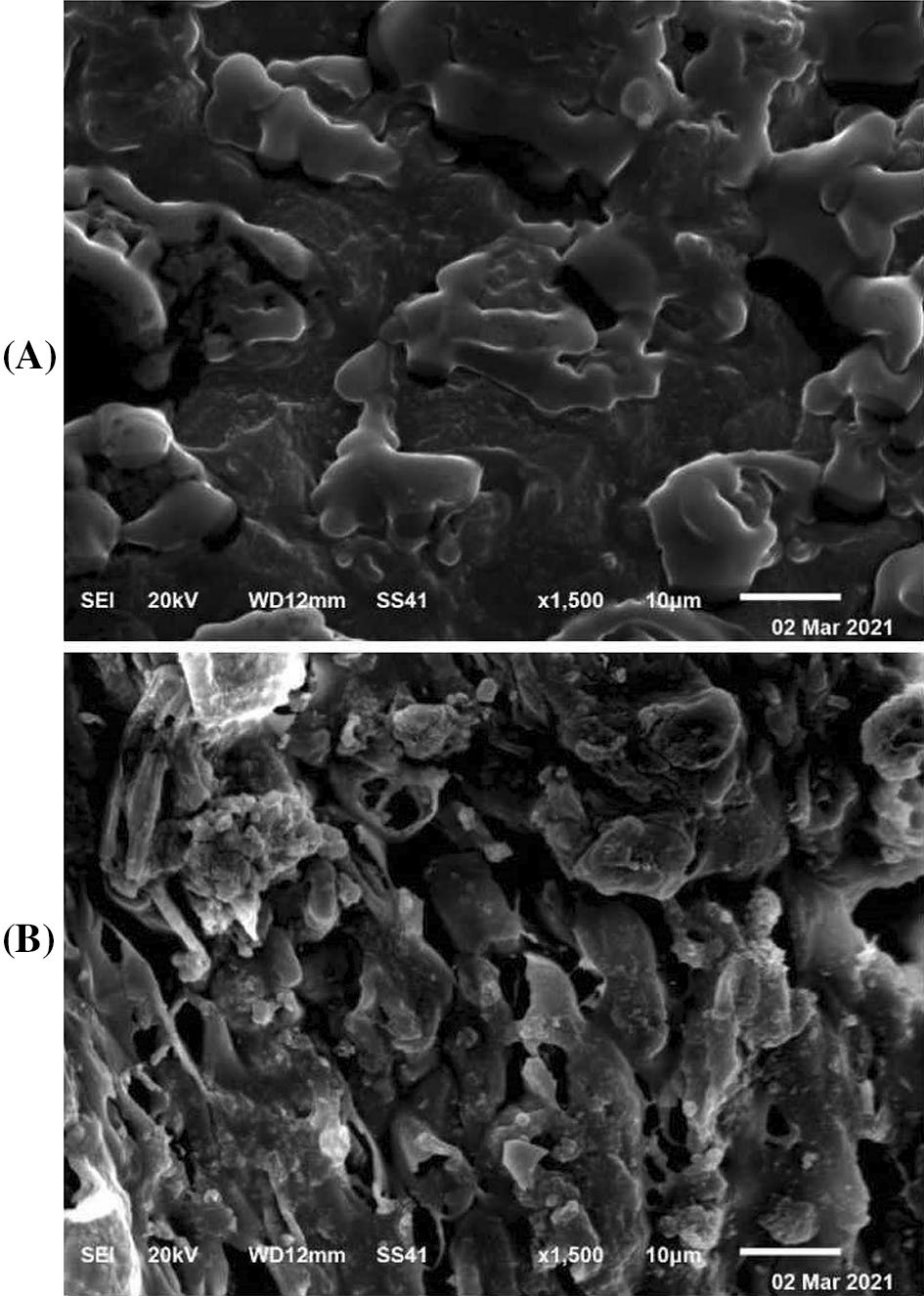
Scanning electron micrographs of *Sargassum latifolium*, showing the control of untreated algal biomass (**A**), and algal biomass treated with Cobalt(II) and MB (**B**).

The scanning electron microscopy for investigation of the surface of algal biomass of *S*. *latifolium*, was analyzed before and after biosorption process of Cobalt(II) and MB ions. The depicted data as shown in Fig. 5 A, showed the regularity of surface biomass of alga, *S*. *latifolium*. However, the surface of biomass as shown in the same Fig. 5 B, seemed to be irregular with swelling of the cells. As well as, the surface seemed to be a new mosaic with massive particles and layers. Generally, the change associated with the treated sample owing to a potent binding of active groups of cells of alga with Cobalt(II) and MB ions. These data are comparable with that reported by Rangabhashiyam and Balasubramanian ^1^ who described a mosaic form of algal biomass, owing to some active groups e.g., amide, carboxylate, thiols, hydroxide, etc. that are conscientious for binding with heavy metals.

### FTIR spectra analysis of *S. latifolium* biomass

**Figure 6:**
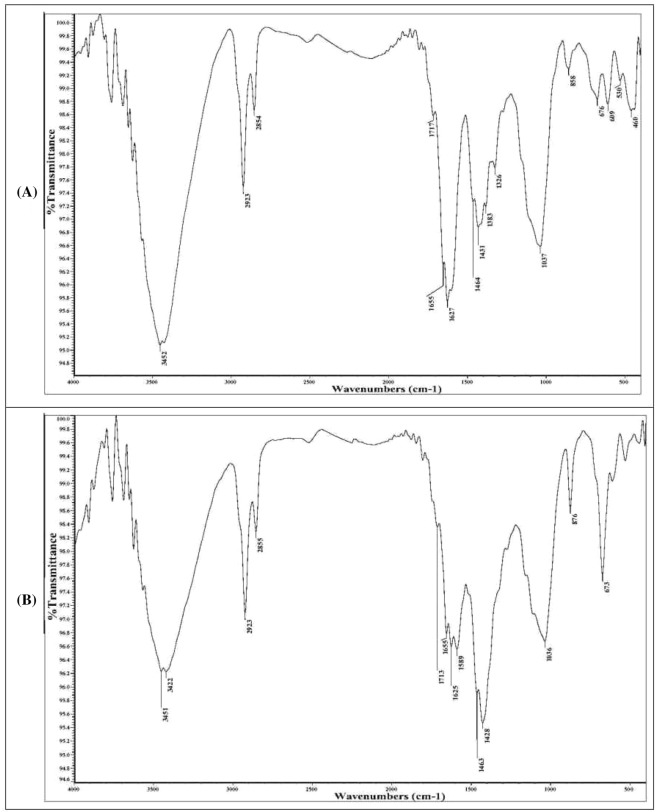
FTIR spectral analysis for *Sargassum latifolium* biomass before (**A**) and after (**B**) dual biosorption of Cobalt(II) and MB dye.

The infrared spectroscopic analyses were performed for two samples including *S. latifolium* alga (control sample), and *S. latifolium* treated simultaneously with cobalt ions and MB (Fig. 6 and Table 5). The IR data related to the characteristic absorption bands are shown in Fig. 6. In accordance, the IR spectroscopic analyses were accomplished to elucidate the functional groups with the investigation of the frequency assigned for a distinct group of the tested samples. The plate of recorded frequencies is in the range of ν = 400 to 4000 cm^−1^ of wavenumbers. The variances of the results recorded for a definite absorption band by shifted value than its value in the control sample, appearance of new absorption bands, and disappearance of characteristic absorption bands in the IR spectra of the two samples. The objective of the IR spectral analyses is to detect the change in the values by shifted or disappeared values to deduce the formed new bonds, owing to the interaction between *S. latifolium* with cobalt ion, and MB dye simultaneously.

**Table 5:** Bands and corresponding functional group of FTIR spectral analysis for *Sargassum latifolium* alga before and after biosorption process.

*Sargassum latifolium* (Control)		*Sargassum latifolium* (Cobalt(II) + methylene blue)	
Wave no. (cm^−1^)	Functional group	Wave no. (cm^−1^)	Functional group
3452	Strong, broad O–H stretching	3451, 3422	Strong, broad O–H stretching
2923	Strong, broad N–H stretching or medium C–H stretching	2923	Strong, broad N–H stretching or medium C–H stretching
2854	Medium C–H stretching, aldehyde	2855	Medium C–H stretching, aldehyde
1717	Strong C=O stretching, α,β-unsaturated ester	1713	Strong C=O stretching, α,β-unsaturated ester
1655	Strong C=O stretching, δ-lactam	1655	Strong C=O stretching, δ-lactam
1627	Strong C=C stretching, α,β-unsaturated ketone	1625	Strong C=C stretching, α,β-unsaturated ketone or C = N stretching
		1589	Strong N–O stretching
1464	Medium C–H bending	1463	Medium C–H bending
1431	Medium O–H bending, carboxylic acid	1428	Medium O–H bending, carboxylic acid
1383	Strong S=O stretching		
1326	Medium O–H bending, phenol		
1037	Strong S=O stretching, sulfoxide	1036	Strong C=S stretching
858	Strong C–H bending, 1,3-disubstituted	876	Strong C–H bending, 1,3-disubstituted
676	Strong C=C bending, alkene	673	Strong C=C bending, alkene
609	C–H bending		
530, 460	L–X, X=halogen or Si–O stretching		

Accordingly, the results of the two samples demonstrated that distinctive absorption bands recorded at ν = 3422–3453 cm^−1^ are attributed to the strong stretching vibration of broad O–H “hydroxy” groups. In addition, the absorption bands at ν = 2923, and 2854–2855 cm^−1^ are assigned to strong stretching broad “N–H” or medium stretching “C–H”, and “C–H” “aldehyde” groups without any observable change between these values in the two samples. A characteristic absorption band at ν = 1717 cm^−1^ in the IR spectrum of *S. latifolium*, is due to the stretching vibration of strong C=O “α, β-unsaturated ester”, this band appeared in the IR analysis of the *S. latifolium* treated simultaneously with cobalt ions and MB dye at ν = 1713 cm^−1^ with −4 shifted value. The same sequence was noticed for the absorption band recorded at ν = 1655 cm^−1^ for *S. latifolium* which is attributed to the strong stretching vibration of C=O group “*δ*-lactam”. This value has appeared in the IR analysis of *S. latifolium* biomass treated simultaneously with cobalt ions and MB dye. The absorption band at ν = 1627 cm^−1^ is attributed to the strong stretching vibration of C=C group “*α*,*β*-unsaturated ketone”, this value was also recorded in the IR analysis of the treated samples at ν = 1624–1628 cm^−1^ but with shifted values by + 1, and −3. Additionally, new absorption bands of strong stretching vibration owing to N–O groups were recorded in the treated sample with Co and MB simultaneously at ν = 1544–1593 cm^−1^, although this band has not appeared in the analysis of the control sample.

On the other hand, the frequency of medium C–H bending groups was recorded for the two samples with shifted values in the range of ν = 1463 to 1466 cm^−1^. It was noticed that the absorption band due to the strong stretching band of the S=O group at ν = 1383 cm^−1^ disappeared in the IR analyses of the sample of control treated with cobalt ions, and methylene blue. The characteristic absorption band at ν = 1326 cm^−1^ is attributed to medium binding vibration of the O–H phenolic group, this value was not recorded for the treated sample with cobalt ions, and MB dye.

The absorption bands due to the strong stretching vibration of S=O group “sulfoxide” are invariable retained at ν = 1034–1037 cm^−1^ with shifted values at −1 to −3 cm^−1^. The characteristic absorption band due to bending C–H group “1,3-disubstituted” at ν = 858 cm^−1^ in the IR spectral analysis of the control sample, at ν = 876 cm^−1^ in the control sample treated with coupled cobalt ion and MB dye. The strong bending vibrations of the identified “C=C” alkene groups were recorded in the IR spectra of all samples at ν = 676 cm^−1^ for the control sample with shifted values by −4, and −3 cm^−1^ in the treated sample.

It was tediously mentioned that the frequency due to bending C–H group, the band recorded values in the range of ν = 609–612 cm^−1^ for the control and treated sample. The frequency values at ν = 530 and 460 cm^−1^ are attributed to the halogen bond in the control sample or stretching vibration due to the Si–O group. The IR spectral analysis at ν = 403 cm^−1^ showed an absorption band, not recorded for the *S. latifolium* treated with MB dye and cobalt ion. The reported studies of FTIR analysis showed that favorable surface properties and the active functional groups play an important role in enhancing biosorption of Cd and Zn ions onto the brown alga *Sargassam polycystum* during the biosorption of metals, Cd and Zn^69^. FTIR illustrated that amino, hydroxyl, and carbonyl functional groups played an important role in the biosorption of both MB and Pb(II) onto the biomass of *Sargassum muticum* brown alga. FTIR analyses clear changes in the features of the algal biomass as a result of the biosorption process of simultaneous bioremoval of Cu^2+^ ions and MO dye by brown alga *Fucus vesiculosus*^32^. Kaplan^70^, and Monteiro et al.^71^ found that the functional groups of algal biomasses involved amino, carboxyl, thioether, sulfhydryl, phosphate, phenolic, and nitrogen of the peptide bond and amide moieties. The other functional groups were found to be teichuronic acid, peptidoglycan, and teichoic acid, and the other biomolecules were polyelectrolytes in nature, with specifically charged groups^1^.

## Conclusion

Taguchi design was applied exclusively in the current study and was found to model the biosorption process of both Cobalt(II) and MB individually, with reasonable values of R^2^ and adjusted R^2^. The independent variables i.e., initial doses of Cobalt(II), and MB, as well as algal biomass, contact time, and pH, play a crucial role in biosorption process of the two responses. To our knowledge, and for the first time, the hybrid ANN was found to be efficient in simultaneous modeling of both Cobalt(II) and biosorption. The experimental values of responses were comparable to other predicted values. SEM photographs of biomass surface showed to be mosaic architecture, with swelling and massive particles in treated samples. As well, the FTIR analysis showed several functional groups with participation in binding both Cobalt(II) and MB ions, such as hydroxyl, aromatic amines, α-β-unsaturated ester, N–O, and α-β-unsaturated ketone. Ultimately, the hybrid artificial neural network showed to be more efficient with accurate optimization of sorption of both Cobalt(II) and MB dye simultaneously, by *S*. *latifolium*. Generally, the biosorption of heavy metals and MB dye by algal biomass could not be comparable with the other biosorbent agents, by which the algal biomass has many merits like as higher affinity with binding of metals and dyes, economy, higher efficiency and availability of wide biomass, as well as, it could be offered a generous opportunity as low-cost biosorbent for therapy of industrial effluents.

## Data Availability

All data generated or analyzed during this study are included in this published article.
